# Self-sampling and self-testing for HIV at a commercial and community-based test provider in the Netherlands: user preferences and usability

**DOI:** 10.1186/s12913-025-12252-4

**Published:** 2025-01-25

**Authors:** I. J. M. Willemstein, O. Shobowale, A. M. Żakowicz, H. Bos, E. L. M. Op de Coul

**Affiliations:** 1 Centre for Infectious Disease Control, National Institute for Public Health and the Environment, P.O. Box 1, Bilthoven, 3720 BA The Netherlands; 2 AIDS Healthcare Foundation (AHF) Europe, Amsterdam, the Netherlands; 3 Soa Aids Nederland, Amsterdam, the Netherlands

**Keywords:** HIV, HIV testing, Self-testing, HIV infections, Population characteristics, Consumer behavior

## Abstract

HIV self-sampling and -testing (HIVSS/ST) reduces testing barriers and potentially reaches populations who may not test otherwise. In the Netherlands, at-home HIV tests became commercially available around 2016, but data on user experiences are limited. This study aimed to explore characteristics of users and their experiences with HIVSS/ST. In 2022 and 2023, a survey was distributed among users of one online commercial provider and one community-based provider; either users ordered a commercial online HIVSS/ST or they sought a free-of-charge HIVST via the community-based provider. Questions included usability, preferences, and barriers of HIVSS/ST-testing. We compared characteristics and experiences of users between providers, risk groups and first-time and repeat testers. In total, 133 users completed the survey; 89 (67%) via the commercial provider and 44 (33%) via the community-based provider. Response rates per provider were 2% and 26%, respectively. Users who bought their test at the commercial provider were more often Gay and Bisexual Men (GBM) (42%), 35 + years (43%), and born in the Netherlands (89%), compared to those recruited through the community-provider (respectively 27%, 25%, 14%). GBM were more often repeat and recent HIVSS/ST-testers, and using pre-exposure prophylaxis (PrEP). Women and heterosexual men were more likely to buy an HIVSS/ST as part of a combination Sexually Transmitted Infections (STI) test package. Overall, main reasons for choosing HIVSS/ST were saving time (42%), anonymity (36%) and not having to talk to a GP (35%). Twenty-two percent of the study participants experienced some problems performing the HIVSS/ST, the most reported problem was obtaining sufficient blood through the finger prick (71%). Recommendations to improve accessibility of HIVSS/ST included more awareness (advertising by trustful providers), more access locations (pharmacies/supermarkets/schools) and lower costs. Our findings indicate that HIVSS/ST is a valuable additional HIV testing method for users, but more insight into the contribution of HIVSS/ST to HIV prevention policies is needed.

**Supplementary Information:**

The online version contains supplementary material available at 10.1186/s12913-025-12252-4.

## Introduction

At home HIV self-sampling (HIVSS) and self-testing (HIVST) may provide an important contribution to reaching the 95–95-95 target set by Joint United Nations Programme on HIV/AIDS (UNAIDS) [1]. It is recommended by the World Health Organization (WHO) to reach people who may not test otherwise, as well as to increase frequency of HIV testing among people with ongoing risk of HIV [2]. HIVSS or HIVST (hereafter referred to as HIVSS/ST) allows a person to privately collect a blood or oral fluid specimen, perform the test, and either interpret the test result by themselves (self-test) or send it to a laboratory for the result (self-sample) [2, 3]. In general, HIVSS/ST have a high sensitivity and specificity that slightly depends on type of test (oral vs blood), although HIVST are sometimes unable to detect early infections in the past few weeks [4]. Barriers to test, such as travel distance, costs, privacy, confidentiality and stigma can be overcome with self-collected testing at home [5, 6].

In the Netherlands, 388 new HIV diagnoses were reported in 2023; 62% among Gay and Bisexual Men (GBM). The majority (92%) of the new diagnoses was identified at regular test sites, such as Sexual Health Centers (SHCs), general practitioners (GPs), and hospitals [7]. HIVSS/ST kits became commercially available around 2016, and availability has been expanded in 2019 to pharmacies and community-based settings [8]. Commercially available HIVSS/ST in the Netherlands started with two Dutch websites that sold HIVST in 2016 [9], up to more than 20 online HIVSS- and HIVST-providers found by the authors in 2023. Although only a couple of these websites fulfill the quality selection criteria for HIVSS/ST as determined by STI AIDS Netherlands [10]. The introduction of HIVSS/ST at Dutch pharmacies in 2019 was nationwide, although pharmacies offering HIVSS/ST are more often located in the highly-urbanized areas [8]. Furthermore, community-based initiatives were set up in the Netherlands (mainly in Amsterdam and Rotterdam) to provide HIVST [11]. In Dutch SHCs self-collection of samples for HIV has not yet officially been implemented, but a pilot implementation study among GBM is performed in parts of the Netherlands [12].

As HIVSS/ST in the Netherlands are mainly offered anonymously and by organizations other than SHCs, data on the characteristics and experiences of users is scarce. Studies in the Netherlands about self-testing often report on intention to use HIVST [12], or specific groups of users (i.e. non-western migrants and/or GBM) [12–14]. In other European countries, availability of data about HIVSS/ST depends on the testing policy. HIVSS/ST testing policy and legislation per country is of influence on HIVST usage and results of other countries are therefore hardly comparable to the Netherlands. In the Netherlands, commercial HIVST have been introduced since 2016, but the provision remained under supervision of a pharmacist or doctor until 2022, when the so-called “kanalisatieregeling” was abolished, allowing commercial (non-medical) organizations to offer these tests as well [15]. Following this change, the HIVSS/ST offer in the Netherlands further increased. For this reason, more insight on users at different test providers is needed. This information could inform the Dutch HIV testing policy and help to improve HIVSS/ST services fit with users’ needs.

To gain insight into HIVSS/ST users at different test providers in the Netherlands, we explored characteristics of users and their experiences with HIV self-sampling or -testing at an online commercial and a community-based free-of-charge test provider in the Netherlands.

## Methods

### Data collection

From April 2022 to June 2023, a survey link was distributed among users who ordered a commercial HIVSS (Soapoli-online (SPO) laboratory test) or a HIVST (Simplitude™ ByMe™) online (either a single HIV test or as part of an STI combination test package with prices ranging from 33 euros for an HIVST up to 125 euros for an HIVSS/STI combination package), or a free-of-charge HIVST (INSTI®) via a community-based provider (AHF Checkpoint Amsterdam) in the Netherlands.

At the commercial provider, individuals could order test kits online by themselves, with the help of an online tool to assess the risk for STI and HIV in order to choose the right test. Personal advice and assistance by nurses and/or doctors was optional. The advice option could be requested via WhatsApp, email, telephone- or video call at any time in the process. The result of the HIVSS was communicated via an online result portal. In the event of a reactive HIV result, individuals were invited by email for a telephone consultation with a health care professional to offer personal support and referral to appropriate care through direct guidance. At the community-based test provider HIVST were also mainly offered online, promoted by posts on social media, their website, flyers in community spaces, and by informing individuals visiting outreach locations of this provider. All contact with users was conducted by WhatsApp, email or telephone and additional so-called “hotline support” was provided at all stages. Before the HIVST kit was sent to the user, a pre-test consultation via video or phone call (mostly phone call) was planned including pre-test counseling, linkage to relevant prevention services (e.g. Post-Exposure Prophylaxis, Pre-Exposure Prophylaxis (PrEP), testing for other STI), and onsite and/or assisted testing if the client preferred. Users were asked to send the result of the HIVST via a photo on WhatsApp within a week after HIVST delivery. If this is not done, 3× weekly WhatsApp reminders were sent. In the event of a reactive result, linkage to care was provided, depending on clients’ personal situation and preference (through a GP, directly to the HIV treatment clinic, or through ‘Kruispost’, a health center that provides medical and social care for people with limited access to regular healthcare services including undocumented migrants or people without health insurance).

All HIV tests obtained blood via a finger prick. INSTI® is an HIV-1/HIV-2 antibody test that yields the test result within one minute and ByMe within 15 minutes. For HIVST, a window period of 12 weeks between last sexual contact and testing is recommended by both providers. The HIVSS used at the commercial provider provides a test result based on 4th generation HIV test (P24-Antigen and HIV-1/2 antibodies). The overall turnaround time of this test is at a maximum of two working days (i.e. blood samples that arrive on working days are analyzed the same day). Positive results are confirmed by Western Blot, taking a maximum of four working days. For this self-sample, a window period of four to six weeks is recommended. All HIV tests have a sensitivity of 99.6–99.9% and specificity of 99.5–99.9% [15, 16].

Users of HIVSS/ST were eligible for this study if aged 18 years and older, and able to understand Dutch or English as the survey was distributed in both languages. At the commercial provider, users who bought an HIVSS or HIVST received an invitation link to the survey along with their test result. At the community-based provider, a flyer with a QR-code with link leading to the survey was distributed with the HIVST package. People who used the QR code or survey link were directed to an introductory screen with information about the objective of the study, the duration of filling in the survey (10 minutes), its guarantee of anonymity, the provision of an incentive, and a participant information statement. Participants provided informed consent before continuing to the questions. As an incentive, a 10 euro gift card was offered for participation after completion of the questionnaire.

After completing the informed consent, participants were linked to the questionnaire in Formdesk. The questionnaire was based on two previous studies on HIVSS/ST at pharmacies and via social networks in the Netherlands [8, 17], and adapted to the goals of this study (Supplementary File S1). The survey was pre-tested by researchers from the National Institute for Public Health and the Environment (RIVM) and employees at the two collaborating test providers. The questionnaire included 35 questions (pre-coded or open), with some adaptations for each provider (e.g. type of test and delivery method). Characteristics from participants collected were: age group, gender (man, woman, transgender/non-binary/other), and sexual orientation (i.e. all women, heterosexual men, GBM (transgender persons were not included in GBM category, including those (1) whose sexual partners are men and (2) whose sex assigned at birth was male or current gender is ‘man’), relationship status, country of birth, country and province of residence, and education level. Participants were also asked about PrEP use (ever used, current use, and daily vs event-driven), HIV sexual transmission risk, HIV testing history and preferences, reasons to choose for HIVSS/ST, HIVSS/ST test type, experiences with obtaining and performing HIVSS/ST, the result of the HIVSS/ST, and recommendations to improve testing services.

### Statistical analyses

In descriptive analyses, characteristics of users and their experiences with HIVSS or HIVST from commercial and community test services were compared. Subsequently, results of women and heterosexual men (combined) were compared with GBM (gender diverse persons excluded), and first-time-testers were compared with repeat testers. As all questions in the survey were compulsory, no data were missing. To assess significant differences between groups, Chi-squared (X^2^) tests were used with Yates continuity correction to compensate for small numbers [18]. In case of small numbers per category (more than 20% of the expected cell counts below 5) the Fisher’s Exact test was applied. A *p*-value of 0.05 was considered a significant difference between groups. Analyses were performed in R studio (version 4.3.1).

### Ethics statement

This study was assessed by The Medical Ethics Committee of the Amsterdam Medical Center as not subjected to the Dutch law for Medical Research Involving Human Subjects Act (WMO) (W21_132 #21.147). All participants gave informed consent before participating in this study.

## Results

### Study population

In total, 4,185 survey invitations were sent and 3.2% (*n* = 133) completed the survey. Invitations were sent to 3,507 users who ordered a commercial HIVSS online, to 516 users who ordered a commercial HIVST online, and to 168 users who received their HIVST from the community-based test provider. Respectively 88 (2.5%), 1 (0.2%) and 44 (26.2%) users completed the survey. Of the 133 users completing the survey, 89 (66.9%) participated via the commercial provider and 44 (33.1%) via the community-based provider (Table 1). In total, most participants were GBM (36.8%) and women (35.3%), aged 25–34 years old (43.6%), living (98.5%) and born (63.9%) in the Netherlands, and were highly educated (77.4%). Survey participants recruited through the commercial provider were significantly more often 35 years or older (vs ≤ 34 years, *X*^2^ = 9.726, *df* = 2, *p* = 0.008), living in a village (vs a city, *X*^2^ = 4.911, *df* = 1, *p* = 0.027) and were born in the Netherlands (vs born abroad, *X*^2^ = 68.830, *df* = 1, *p* < 0.001). Although not significant, more women and heterosexual men used the HIVST at the community-based provider compared to GBM (*X*^2^ = 1.434, *df* = 1, *p* = 0.231, Supplementary Table S2), which was the largest group participating via the commercial provider. Repeat users were significantly more often GBM, compared to first-time users (Fisher’s Exact test *p* = 0.007, Supplementary Table S3). Other differences in characteristics of GBM and women and heterosexual men and first-time and repeat-testers were non-significant (Supplementary Table S2 & S3).

**Table 1:** Characteristics of study participants using HIVSS/ST at an online commercial/community provider in the Netherlands

	Total	Online commercial provider	Community-based provider	Test statistic	
	n (%)	n (%)	n (%)	X^2^ (df) ^a^	*p*-value
Total	133 (100)	89 (100)	44 (100)		
Gender and sexual orientation				-	0.102
Women	47 (35.3)	28 (31.5)	19 (43.2)		
Heterosexual men	33 (24.8)	23 (25.8)	10 (22.7)		
GBM	49 (36.8)	37 (41.6)	12 (27.3)		
Transgender/non-binary/other	4 (3.0)	1 (1.1)	3 (6.8)		
Age group				9.726 (2)	**0.008**
18–24	26 (19.5)	11 (12.4)	15 (34.1)		
25–34	58 (43.6)	40 (44.9)	18 (40.9)		
35 +	49 (36.8)	38 (42.7)	11 (25.0)		
Country of residence					
The Netherlands	131 (98.5)	88 (98.9)	43 (97.7)	-	1.000
Other	2 (1.5)	1 (1.1)	1 (2.3)		
Province of residence^b^					
North (Drenthe, Groningen, Friesland)	7 (5.3)	3 (3.4)	4 (9.3)	-	0.427
East (Flevoland, Overijssel, Gelderland)	16 (12.2)	11 (12.5)	5 (11.6)		
South (Noord-Brabant, Limburg)	25 (19.1)	19 (21.6)	6 (14.0)		
West (Noord-Holland, Zuid-Holland, Utrecht, Zeeland)	83 (63.4)	55 (62.5)	28 (65.1)		
Place of residence^b^					
City	95 (72.5)	58 (65.9)	37 (86.0)	4.911 (1)	**0.027**
Village	36 (27.5)	30 (34.1)	6 (14.0)		
Country of birth				68.830 (1)	** < 0.001**
The Netherlands	85 (63.9)	79 (88.8)	6 (13.6)		
Other	48 (36.1)	10 (11.2)	38 (86.4)		
Education level				2.281 (1)	0.131
≥ Higher vocational education or University	103 (77.4)	65 (73.0)	38 (86.4)		
≤ Intermediate vocational education	30 (22.6)	24 (27.0)	6 (13.6)		
Relationship status				-	0.609
Single	50 (37.6)	36 (40.4)	14 (31.8)		
Dating	47 (35.3)	28 (31.5)	19 (43.2)		
Steady partner	30 (22.6)	21 (23.6)	9 (20.5)		
Other	6 (4.5)	4 (4.5)	2 (4.5)		
Type HIV test				124.2	** < 0.001**
HIV self-test	45 (33.8)	1 (1.1)	44 (100)		
HIV self-sample	88 (66.2)	88 (98.9)	NA		

*GBM* Gay and Bisexual men, *NA* not available

^a^If more than 20% of the expected cell counts were below 5, Fisher’s exact test was applied instead of chi-square test

^b^Percentage of persons living in the Netherlands

None of the participants reported a reactive HIV test (95.5% HIV negative, 4.5% unknown, Table 2). The majority of participants (66.9%, *n* = 89) had tested for HIV before of whom 43.8% had tested in the past 6 months. GBM tested significantly more often previously (83.7%) and in the past 6 months (56.1%), compared to women and heterosexual men (56.3% respectively 35.6%, Supplementary Table S4). A small proportion of the participants (6.8%, *n* = 9, all were GBM and repeat users) used or had used PrEP of whom 7 (77.8%) reported current use (Supplementary Table S4 & S5). Finally, at the commercial provider, GBM more often ordered an HIVSS only compared to women and heterosexual men who often ordered an HIVSS as part of an STI combination test package (Chlamydia, Gonorrhea, (Trichomoniasis), syphilis and HIV) (Fisher’s Exact test *p* = 0.009, Supplementary Table S4).

**Table 2:** HIV-related characteristics of study participants using HIVSS/ST at an online commercial/community provider in the Netherlands

	Total	Online commercial provider	Community-based provider	Test statistic	
	n (%)	n (%)	n (%)	X^2^ (df) ^a^	*p*-value
Total	133 (100)	89 (100)	44 (100)		
HIV test result				-	0.093
HIV negative	127 (95.5)	87 (97.8)	40 (90.9)		
HIV positive	0 (0.0)	0 (0.0)	0 (0.0)		
Unknown^b^	6 (4.5)	2 (2.2)	4 (9.1)		
Ever tested for HIV				-	0.836
No	44 (33.1)	30 (33.7)	14 (31.8)		
Yes, 1–2 times	48 (36.1)	30 (33.7)	18 (40.9)		
Yes, 3–4 times	15 (11.3)	10 (11.2)	5 (11.4)		
Yes, 5 times or more	26 (19.5)	19 (21.3)	7 (15.9)		
When was last HIV test?^c^				-	0.169
In the last 6 months	39 (43.8)	28 (47.5)	11 (36.7)		
6–12 months ago	18 (20.2)	9 (15.2)	9 (30.0)		
More than 1 year ago	31 (34.8)	22 (37.3)	9 (30.0)		
I don't remember	1 (1.1)	0 (0.0)	1 (3.3)		
Ever used PrEP				-	0.271
No	124 (93.2)	81 (91.0)	43 (97.7)		
Yes	9 (6.8)	8 (9.0)	1 (2.3)		
Condomless sex past 3 months				**-**	**0.022**
No	37 (27.8)	21 (23.6)	16 (36.4)		
Yes	94 (70.7)	68 (76.4)	26 (59.1)		
I would rather not say	2 (1.5)	0 (0.0)	2 (4.5)		
Condomless sex last intercourse				-	0.466
No	48 (36.1)	33 (37.1)	15 (34.1)		
Yes	82 (61.7)	55 (61.8)	27 (61.4)		
I would rather not say	3 (2.3)	1 (1.1)	2 (4.5)		
Type of HIVSS/ST^d^				-	-
Combination STI SS package (Chlamydia, Gonorrhea, (Trichomoniasis), syphilis and HIV)	40 (30.1)	40 (44.9)	NA		
Syphilis- and HIV-SS	19 (14.3)	19 (21.3)	NA		
HIVSS	29 (21.8)	29 (32.6)	NA		
HIVST	1 (0.8)	1 (1.1)	NA		
How obtained HIVSS/ST^d^				-	-
Package delivery	9 (6.8)	9 (10.1)	NA		
Letter post delivery	61 (45.9)	61 (68.5)	NA		
Pick-up at package point	13 (9.8)	13 (14.6)	NA		
Pick-up at regular pharmacy (not online)	5 (3.8)	5 (5.6)	NA		
Pick-up at 24-h vending machine	1 (0.8)	1 (1.1)	NA		
Performed HIVSS/ST				-	0.254
No	3 (2.3)	1 (1.1)	2 (4.5)		
Yes	130 (97.7)	88 (98.9)	42 (95.5)		
If not, why				-	0.333
I didn’t have the time yet	2 (66.7)	0 (0.0)	2 (100.0)		
I forgot	1 (33.3)	1 (100.0)	0 (0.0)		
Problems performing HIVSS/ST^e^				2.617 (1)	0.106
No	102 (78.5)	65 (73.9)	37 (88.1)		
Yes	28 (21.5)	23 (26.1)	5 (11.9)		
Would you know what to do if you got an HIV positive ST?				**-**	**0.026**
No	41 (30.8)	32 (36.0)	9 (20.5)		
Yes	90 (67.7)	57 (64.0)	33 (75.0)		
I would rather not say	2 (1.5)	0 (0.0)	2 (4.5)		

*SS* self-sample, *ST* self-test, *STI* sexually transmitted infections, *NA* not available

^a^If more than 20% of the expected cell counts were below 5, Fisher’s exact test was applied instead of chi-square test

^b^Unknown include persons who would rather not say the result, have not looked for the results or have not taken the test yet and persons of whom the test failed

^c^Percentage of persons ever tested for HIV

^d^Only included in questionnaire of commercial provider

^e^Percentage of persons who performed the HIVSS/ST

### User preferences and usability of HIV self-samples and -tests

Of the 133 users of HIVSS/ST, 101 (75.9%) heard about the HIVSS/ST via internet (Table 3). GBM significantly more often heard about HIVSS/ST via Sexual Health Centers (SHCs) (Fisher’s Exact test *p* = 0.010, Supplementary Table S6). Among those testing via the commercial provider (*n* = 89), the main reason for choosing HIVSS/ST was not having to talk to a GP about HIV tests (38.2%, *n* = 34), followed by saving time (36.0%, *n* = 32) and waiting lists at the SHCs (30.3%, *n* = 27). Among those testing via the community-based provider (*n* = 44), free-of-charge testing (95.5%, *n* = 42), immediate result(s) (65.9%, *n* = 29) and saving time (54.5%, *n* = 24) were the most often reported reasons for choosing HIVST. Waiting lists at SHCs was reported by 13.6% of the users (*n* = 6) via the community-based provider to be a reason for choosing HIVST. For both providers, anonymity was also often reported as reason for choosing HIVSS/ST (28.1% at commercial provider and 52.3% at community-based provider). Also, for the users at the community-based provider HIVST-use was more often reported because users did not have a GP to test at (20.5% vs 1.1%, Fisher’s Exact test *p* < 0.001). Reasons to test for GBM and women and heterosexual men and first-time and repeat testers showed no significant differences (Supplementary Tables S6 & S7).

**Table 3:** Usability and preferences of study participants using HIVSS/ST an online commercial/community provider in the Netherlands

	Total	Online commercial provider	Community-based provider	Test statistic	
	n (%)	n (%)	n (%)	X^2^ (df) ^a^	*p*-value
Total	133 (100)	89 (100)	44 (100)		
How did you hear about HIVSS/ST?					
Pharmacy	0 (0.0)	0 (0.0)	0 (0.0)	-	-
GP	1 (0.8)	1 (1.1)	0 (0.0)	-	1.000
Public health services (GGD) or SHC	12 (9.0)	12 (13.5)	0 (0.0)	-	**0.009**
Previous HIVSS/ST	19 (14.3)	16 (18.0)	3 (6.8)	2.152 (1)	0.142
A friend	11 (8.3)	3 (3.4)	8 (18.2)	-	**0.006**
Internet	101 (75.9)	70 (78.7)	31 (70.5)	0.681 (1)	0.409
Social media	2 (1.5)	0 (0.0)	2 (4.5)	-	0.108
Main reasons for HIVSS/ST at online commercial or community-based provider					
It is cheap^b^	10 (7.5)	10 (11.2)	NA	-	-
It is for free^c^	42 (31.6)	NA	42 (95.5)	-	-
Good previous experiences with online order of HIV or STI self-tests	16 (12.0)	14 (15.7)	2 (4.5)	2.504 (1)	0.114
Good previous experiences with this commercial test provider^b^	25 (18.8)	25 (28.1)	NA	-	-
Good previous experiences with this community provider^c^	5 (3.8)	NA	5 (11.4)	-	-
Does not require to talk to my GP about HIV tests	47 (35.3)	34 (38.2)	13 (29.5)	0.624 (1)	0.430
I don’t have a GP	10 (7.5)	1 (1.1)	9 (20.5)	-	** < 0.001**
Anonymity	48 (36.1)	25 (28.1)	23 (52.3)	6.454 (1)	**0.011**
No GGD/SHC nearby	11 (8.3)	9 (10.1)	2 (4.5)	-	0.337
No (soon) open spots available at GGD/SHC	33 (24.8)	27 (30.3)	6 (13.6)	3.552 (1)	0.060
Don’t know any other way to test for HIV	8 (6.0)	2 (2.2)	6 (13.6)	-	**0.016**
Prefer a finger prick test, because I don’t want blood drawn	5 (3.8)	3 (3.4)	2 (4.5)	-	1.000
Immediate results	34 (25.6)	5 (5.6)	29 (65.9)	53.122 (1)	** < 0.001**
It saves time	56 (42.1)	32 (36.0)	24 (54.5)	3.447 (1)	0.063
Information used with performing HIVSS/ST^d^					
On website of test provider	28 (21.5)	23 (26.1)	5 (11.9)	2.617 (1)	0.106
The paper manual that came with the HIV test	119 (91.5)	85 (96.6)	34 (81.0)	-	**0.005**
Instruction videos on website of test provider	25 (19.2)	16 (18.2)	9 (21.4)	0.041 (1)	0.840
Instruction videos on another website	7 (5.4)	2 (2.3)	5 (11.9)	-	**0.035**
No information used	1 (0.8)	0 (0.0)	1 (2.4)	-	0.323
If experienced problems performing HIVSS/ST, what problems^e^					
Steps for performing the test too complicated	0 (0.0)	0 (0.0)	0 (0.0)	-	-
Instructions with test were not clear	4 (14.3)	4 (17.4)	0 (0.0)	-	1.000
Forgot one step when performing test	0 (0.0)	0 (0.0)	0 (0.0)	-	-
Afraid of performing finger prick	6 (21.4)	5 (21.7)	1 (20.0)	-	1.000
Afraid of blood with finger prick	0 (0.0)	0 (0.0)	0 (0.0)	-	-
Not enough blood from finger prick	20 (71.4)	16 (69.6)	4 (80.0)	-	1.000
No results / unclear test result	1 (3.6)	0 (0.0)	1 (20.0)	-	0.179
Don’t know how to read the test result	1 (3.6)	1 (4.3)	0 (0.0)	-	1.000

*GP* general practitioner, *SHC* Sexual Health Center, *NA* not available

^a^If more than 20% of the expected cell counts were below 5, Fisher’s exact test was applied instead of chi-square test

^b^Only included in questionnaire of commercial provider

^c^Only included in questionnaire of community-based provider

^d^Percentage of persons who performed the HIVSS/ST

^e^Percentage of persons who performed the HIVSS/ST and experienced problems

Almost all users used the paper manual to perform the HIV test (91.5%, *n* = 119). Online information of the test provider, either on their website (21.5%, *n* = 28) or via instruction videos (19.2%, *n* = 25) were less often used (Table 3). Of all study participants who performed the HIVSS/ST (97.7%, *n* = 130), 28 (21.5%) experienced some problems in performing the test; women and heterosexual men (27.3%) more often than GBM (12.2%), although not significantly (*X*^2^ = 3.174, *df* = 1, *p* = 0.075, Table 2 & Supplementary Table S4). Twenty-three (26.1%) users at the commercial provider reported some problems with the test performance, compared to 5 (11.9%) at the community-based provider (Table 2), mostly obtaining sufficient blood through the finger prick: 16 (69.6%) and 4 (80.0%) at the commercial and community-based provider respectively (Table 3). For both repeat (66.7%, *n* = 12) and first time users (80.0%, *n* = 8) this was the most reported problem (Supplementary Table S7).

### Accessibility of HIV self-samples and -tests

In total, 62 participants (45 at the commercial provider and 17 at the community provider) filled in the open-ended questions asking for recommendations to improve the accessibility of HIVSS/ST at their provider. At the commercial provider, more (online) advertisement was mentioned, in particular “*More visibility on social media, especially on apps such as Grindr and Blued*” was recommended and at the community provider using the words *“free” *and* “anonymous”* were recommended. Another recommendation at the commercial provider was lower costs of tests: “*make it more affordable/cheaper/free-of-charge”*, “*that it is included in the basic insurance”* and “*this should become free; at the GGD [SHC], it’s nearly impossible to schedule an appointment (more demand than capacity)”.* Other recommendations for the community-based provider included more access locations in other cities and “*Add to the info sheet what to do if you are scared of needles (a breathing exercise, 1,2,3, and go, anything else – I found myself hesitating for a few minutes)”*. But also more awareness among GPs about HIVSS/ST, easier access to HIVSS/ST by providing tests at pharmacies/supermarket/schools and better information about trustful HIVSS/ST providers online were reported to improve accessibility of HIVSS/ST.

## Discussion

In this study, we assessed characteristics of HIV self-sampling (HIVSS) or -testing (HIVST) users, and their experiences with HIVSS/ST at different test providers in the Netherlands. Users who bought their test at the commercial provider were more often Gay and Bisexual Men (GBM), 35 + years, and born in the Netherlands. GBM were also more often repeat and recent HIV testers. Women and heterosexual men were more likely to use an HIVSS/ST as part of an STI combination test package. Users experience HIVSS/ST as an anonymous and timesaving way of HIV testing without having to talk to a healthcare professional or as an alternative for waiting lists at the SHCs. Yet some difficulties with performing the finger prick were reported. Recommendations to improve accessibility of HIVSS/ST included more awareness among GPs and through advertising by trustful providers, more access locations (pharmacies/supermarkets/schools) and lower costs.

This is the first study in the Netherlands on characteristics and experiences of HIVSS/ST users, and fills a knowledge gap about this group that can inform HIV prevention policies in the Netherlands. We compared users at different test providers, to assess the value of different providers to HIV prevention. However, there were some limitations. First, the response rate, especially at the commercial provider, was low and overall numbers were small. Therefore, this study lacked statistical power to perform any additional analyses on the comparisons between groups and possible interplays between variables could not be assessed. Also, because of a low sample size, generalizability of the results is limited. It remains unknown to what extent our study participants are representative for all users of HIVSS/ST. However, when comparing our study population at the community-based provider to all users at this provider, we saw a similar distribution of age groups, country of birth and testing history (data not shown). Only in our study the proportion of women was highest (43.2%) compared to other gender and sexual orientation groups, whereas at the provider this proportion was less (34.3%) and the proportion of Men who have Sex with Men (MSM) was highest (36.1%). For the commercial provider characteristics of all users were not available. To ensure anonymity, characteristics of users are not systematically recorded and therefore, data were unavailable for all users. These results should therefore be interpreted with caution, as the results may show a representation of people who fill in a questionnaire rather than people who use HIVSS/ST at the commercial provider. Selection bias might also arise at the commercial provider from distributing the study invitation with the test result. People with an HIV positive result might not want to participate just after receiving this result. Among our respondents, no reactive HIV results were reported, compared to 393 new HIV diagnoses newly reported in care in the Netherlands in 2022 [19], and experiences with HIVSS/ST of people with a newly diagnosed HIV infection and the linkage to care trajectory remain unclear. Finally, it should be noted that the commercial provider sold almost only HIVSS (98.9%) due to active promotion, and the community provider distributed HIVST only. Due to the low number of HIVST distributed via the commercial provider, we could only include one person with a HIVST at this provider in our survey. For this reason, a comparison of experiences of HIVST users between providers could not be made. Future research that considers motivations for the route and type of HIV test chosen is recommended.

Overall, in line with our study, users have positive experiences with HIVSS/ST [12, 20, 21]. HIVSS/ST users experience these tests as easily accessible, highly confidential, quick, convenient and private [4, 20, 22, 23]. Particular reasons for using HIV self-tests were immediate results, to complete the test by oneself, privacy of testing in a place of own choice and ease [4, 24], as was seen in our study at the community-based provider. Other comparisons between HIVSS versus HIVST could not be made based on our study, but as a previous study mentioned no remarkable differences between HIVSS- and HIVST-users [24], we assume that differences in our study rather reflect differences between providers. Concerns raised by those performing the HIVSS/ST are lack of counseling and linkage to care in case of a reactive test result, dislocation from sexual health services, possible user error, accuracy of test results and costs [4, 22, 23, 25]. Costs and preference for including HIVSS/ST in basic insurance was also mentioned in our study. However, in our study, no HIV infections were found. Although this can be a result of selection bias, it might also be that HIVSS/ST users are more repeat testers at low risk (the ‘worried well’) compared to individuals testing at other health facilities. One could argue that HIVSS/ST for this first group is not necessary to prevent HIV infections and therefore adding it to basic insurance or lowering costs may not be cost-effective in the Netherlands. More research is needed to assess whether undiagnosed individuals living with HIV are reached with HIVSS/ST. Performance of HIVSS/ST in our study was, as in other studies [4], without any reported problems by the majority of users. Also a global review of HIVST showed that most users feel confident in reading the test results by themselves [4]. If there was a problem with performing the HIVSS/ST, it was mostly about collecting sufficient blood with the finger prick [4, 20]. In a study comparing blood-based testing with oral-fluid testing, Peck et al. [26] found that the performance of blood-based HIVST is slightly better compared to oral-fluid testing. Yet, if the blood-based test isn’t well performed, the missteps tend to be more severe (e.g. using personal items to perform the finger prick) [20, 26] as was reported in our study as well: *“I heated the needle to make the hole in my finger bigger”.*

To gain insight to what extent HIVSS/ST complements HIV testing by traditional HIV test providers or reaches other groups in the Netherlands, we compared the overall distribution of HIV tests in women, heterosexual men and GBM from our survey (35% women, 25% heterosexual men and 37% GBM) with the percentages of HIV test consultations at SHCs in 2022 [27], and this showed a similar distribution of tests: 31%, 16%, 53% (MSM), respectively, with slightly more women and heterosexual men in our study. This is consistent with literature that female and MSM users tend to use HIV self-sampling-services more often than heterosexuals do [28, 29]. Results regarding age group and self-test usage were inconsistent [28, 29]. In most studies [29] the 35 + age group is less likely to use self-tests compared to younger groups, which is in contrast to our results. However, we did see a significant difference between the community-based and commercial provider; only at the commercial provider most users were 35 + . Possibly, older groups can afford commercial HIV self-tests more easily compared to younger groups. Furthermore, users younger than 25 years of age have free access to SHCs in the Netherlands and may have a preference for these services. Finally, at the community-based provider, users are more often born abroad. This can be explained by the provider's specific aim to reach underserved communities like migrant populations [30]. But differences between providers could potentially reflect that the HIVST via the community-based provider was free-of-charge compared to a paid version at the commercial provider. In a United States’ study looking at willingness to pay among individuals in a community-based program it was found that only 23% of the respondents were willing to pay the market price for HIVST of 40 US dollars [31].

Also, GBM in our survey were comparable to MSM at SHCs, with the largest percentage of consultations with HIV tests among 35 + years (39% in our survey, 42% at SHCs), mostly born in the Netherlands (69% in survey, 60% at SHCs), highly educated (71% in survey, 67% at SHCs) and first-time testers (18% in survey, 15% at SHCs). We found that GBM more often ordered the single HIVSS, whereas heterosexual study participants more often ordered an HIVSS as part of a combination STI test package. Additionally, we found that GBM are most often a frequent and recent self-testing group for HIV. This is in line with a Norwegian study [32] and with the WHO recommendations for key populations such as MSM with certain risk factors (e.g. taking PrEP or visiting SHCs) to test frequently for HIV prevention [33]. This Norwegian study also suggests that HIV self-testing can be an efficient approach in reaching MSM who are rarely or never tested for HIV [32]. In our study, a small proportion of GBM was first-time user. Yet we could not assess if these GBM were at increased risk for HIV and if HIVSS/ST within this group could have amplified HIV prevention in the Netherlands.

As characteristics of our study group show similarities with HIV test consultations at SHCs, one could argue whether our study group of self-testers is the group who may not test otherwise, as aimed by the WHO [1]. Possibly, this group would have tested at SHCs in the Netherlands, if certain barriers such as long waiting lists would not exist. Waiting lists at SHCs was mentioned as a reason to use HIVSS/ST in our study, but this reason may be higher in our study because of COVID-19 measures, less spots were available during and after the pandemic at the SHCs [27]. Our study period began just after the cessation of all measures. Other reasons were that users didn’t want to discuss HIV (risks) with a health care professional (including GPs) or wanted to maintain anonymity. These reasons mark the added value of HIV self-testing in the Netherlands, especially as since the COVID-19 pandemic people are more familiar with self-testing in general. Also, the importance of having a range of HIV testing options was shown in our study. For example, when we compared different types of users (e.g. first-time vs repeat, different sexual preference, differences per provider) within our overall study population, we found that the community provider in our study was able to reach groups other than users who may have tested otherwise, such those born in other countries than the Netherlands. By health promoting and advertising HIVSS/ST at more locations in the Netherlands, but also by active counseling about and providing options by health care professionals, individuals can have a range of options to choose from that fits their needs best.

## Conclusion

Altogether, this study provides a first insight into users’ characteristics and experiences with HIVSS/ST since its availability in the Netherlands. Overall, in our study, HIVSS/ST is experienced as an acceptable and easy to perform HIV testing method. However, users see opportunities to increase accessibility of HIV self-testing and -sampling. As the generalizability of our study is limited, additional research in the Netherlands is needed to better understand what HIVSS/ST-modalities work best for users, so barriers can be overcome and usage can be improved, especially in situations with constraints in access to care, like waiting lists at SHCs, continuing stigma on HIV and financial barriers. Continuous monitoring of HIVSS/ST-usage and diagnoses is recommended to inform HIV prevention and testing policies.

## Supplementary Information

Supplementary Material 1.

## Data Availability

Data are not publicly available due to privacy concerns. The dataset generated and analyzed are available through the corresponding author on reasonable request.
