# Supplementary material for: Self-sampling and self-testing for HIV at a commercial and community-based test provider in the Netherlands: user preferences and usability
Source: BMC Health Serv Res. 2025 Jan 25;25:141. doi: 10.1186/s12913-025-12252-4 (PMC11763118; doi:10.1186/s12913-025-12252-4)
Supplement: Supplementary file 1 — Supplementary Material 1. [file 12913_2025_12252_MOESM1_ESM.docx]

**Supplementary Material**

**Article title: Self-sampling and self-testing for HIV at a commercial and community-based test provider in the Netherlands: user preferences and usability**

**Journal name: BMC Health Services Research**

**Author names:** I.J.M. Willemstein^1*^, O. Shobowale^2^, A. M. Żakowicz^2^, H. Bos^3^, E.L.M. Op de Coul^1^

**Affiliation:**

*^1^ Centre for Infectious Disease Control, National Institute for Public Health and the Environment, Bilthoven, the Netherlands*

*^2^ AIDS Healthcare Foundation (AHF) Europe, Amsterdam, the Netherlands*

*^3^ Soa Aids Nederland, Amsterdam, the Netherlands*

**E-mail address of the corresponding author:**

Eline Op de Coul, eline.op.de.coul@rivm.nl

**Supplementary File S1.** Survey questions

*About you*

1. What is your age group?
   - Younger than 18
   - 18-24
   - 25-34
   - 35-44
   - 45-54
   - 55-64
   - 65 or older
2. What is your gender?
   - Man
   - Woman
   - Transgender/non-binary/other
   - I would rather not say
3. Do you currently live in the Netherlands?
   - Yes
   - No
4. [if Q3 = yes] In which province do you live?
   - Noord-Holland
   - Zuid-Holland
   - Utrecht
   - Flevoland
   - Noord-Brabant
   - Limburg
   - Gelderland
   - Overijssel
   - Drenthe
   - Groningen
   - Friesland
   - Zeeland
5. [if Q3 = yes] Do you live in a city or a village?
   - City
   - Village
6. [if Q3 = no] Which country do you currently live in? [scroll list]
7. What is the highest educational level you have completed or you are currently studying?
   - None
   - Primary school
   - General secondary education (equivalent to MAVO/MULO)
   - Lower vocational secondary education (equivalent to VMBO)
   - Higher general education or A-level/pre-university secondary education (equivalent to HAVO/VWO/Gymnasium)
   - Intermediate vocational education (equivalent to MBO)
   - Higher vocational education or University (equivalent to HBO, undergraduate, postgraduate, PhD)
   - Other, namely
8. Where were you born?

- The Netherlands
- Central Europe (for example Croatia, the Czech Republic, Hungary, Poland, Slovakia, Slovenia)
- Eastern Europe and Central Asia (for example Russia, Ukraine, Lithuania, Estonia, Belarus, Georgia, Moldova)
- Western Europe and North America (The Northern and Western Europe region includes economies from Northern Europe (for example Denmark, Finland, Ireland, Norway, Sweden, and the United Kingdom), and Western Europe (for example Austria, France, Germany, the Netherlands, and Switzerland)
- Southern Europe (for example Albania, Andorra, Bosnia and Herzegovina, Bulgaria, Croatia, Greece, Italy, Malta, Portugal, Romania, San Marino, Slovenia, Spain, Cyprus)
- Sub-Saharan Africa
- North Africa and Middle East (for example Yemen, United Arab Emirates, Tunisia, Syria, Saudi Arabia, Oman, Qatar, Palestine, Morocco, Libya, Lebanon, Kuwait, Jordan, Israel, Iraq, Iran, Egypt, Djibouti, Bahrain, Algeria)
- Latin America
- Caribbean (for example Suriname, Aruba, Curaçao)
- South and South-East Asia
- Oceania (for example Australia or New Zealand)
- Other, namely

1. In terms of sexual orientation, do you identify best as
   - Gay / homosexual
   - Bisexual
   - Heterosexual
   - I would rather not say
   - Other, namely
2. What is your current relationship status?
   - Single
   - Dating someone
   - Dating multiple people
   - I have a steady partner
   - I would rather not say
   - Other, namely

*The following questions are about buying HIV tests* *before and ways to prevent HIV transmission.*

1. Have you ever tested for HIV? *Do not count your current test.*
   - No, never
   - Yes, 1 – 2 times
   - Yes, 3 - 4 times
   - Yes, 5 times or more
   - I don’t know
   - I would rather not say
2. [if Q11 = yes] When was your last HIV test? *Do not count your current test.*
   - In the last 6 months
   - 6-12 months ago
   - 1- 3 years ago
   - More than 3 years ago
   - I don’t remember
3. Where do you prefer to get tested for HIV? *Multiple answers are possible.*

□ At a general practitioner

□ At the public health services (GGD) or STI clinic

□ At an HIV treatment centre (hospital)

□ At the midwife

□ At a laboratory

□ At home (or somewhere else) with a self-test that gives me the test results directly

□ At home (or somewhere else) with a self-sampling kit I have to send to a lab

□ One Day Clinic

□ Testlab (Man tot Man)

□ AHF Checkpoint of AHF Popup location (AIDS Healthcare Foundation)

□ Other, namely

1. In the past 3 months, did you have unprotected sex (anal/vaginal without condom)?

- No
- Yes
- I would rather not say

1. At last sexual intercourse, did you have unprotected sex (anal/vaginal without condom)?
   - No
   - Yes
   - I would rather not say
2. Have you ever used PrEP? *(PrEP is a medicine that people at risk for HIV can take to prevent HIV infection. PrEP stands for pre-exposure prophylaxis)*
   - No
   - Yes
   - I would rather not say
3. [if Q16 = yes] Are you currently using PrEP?
   - No
   - Yes
   - I would rather not say
4. [if Q17 = yes] How do you use PrEP?
   - Daily
   - Before and after sex
   - Other, namely

*You are halfway through the questionnaire.*

*The following questions are about the HIV self-test from at the current provider.*

1. When did you receive/buy the HIV self-test? *If you do not remember this, please try to be as exact as possible.*

Date:

1. What type of HIV self-test did you buy? [for commercial provider only]
   - Combined STD self-sampling test (Chlamydia, Gonorrhea, (Trichomoniasis), syphilis and HIV) that I had to send to the commercial provider for a test result
   - Syphilis and HIV self-sampling test that I had to send to the commercial provider for a test result
   - HIV self-sampling test that I had to send to the commercial provider for a test result
   - ByMe HIV self-test that I could read myself
2. How did you obtain this HIV self-test (or combination HIV/STD self-test)? [for commercial provider only]

- Delivered by package to my home address
- Delivered by letter post to my home address
- Pick-up at a package point
- Pick-up at a regular pharmacy (not online)
- Pick-up at a 24-hour vending machine

1. How did you hear about the HIV self-test from the current provider? *Multiple answers are possible.*

□ Pharmacy

□ General practitioner

□ Public health services (GGD) or STI clinic

□ I previously bought/received an HIV test at this provider

□ Newspaper/magazine

□ Radio or television

□ A friend

□ Online via the website of this provider

□ Online via another website (Google, advertisement website)

□ Social media (Facebook, twitter, Instagram, YouTube)

□ Other, namely

1. What were the main reason(s) you wanted to do an HIV self-test? *Multiple answers are possible.*

□ I was worried about having an HIV-infection
□ Someone close to me (partner/friend/acquaintance) has HIV

□ A bedpartner who has HIV alerted me

□ I wanted to make sure I have no HIV

□ I test every couple of weeks/months to be sure

□ I have had STI before

□ I was involved in ChemSex

□ I was involved in non-consensual sex

□ I feel sick and ill
□ I bought the test for someone else

□ Other, namely

1. What were the main reason(s) to get an HIV self-test from the current provider? *Multiple answers are possible.*

□ It is cheap/for free

□ Good previous experiences with online order of HIV or STI self-tests

□ Good previous experiences with this provider

□ Does not require to talk to my general practitioner about HIV tests

□ I don’t have a general practitioner

□ Anonymity

□ No STI clinic (GGD) nearby

□ No (soon) open spots available at a STI clinic (GGD)

□ I don’t know any other way to test for HIV

□ I would like to do a finger prick test, because I don’t want blood drawn

□ Immediate results

□ It saves time

□ Other, namely

*You are almost done with this questionnaire.*

1. How do you agree with the following statements about receiving the HIV self-test at the current provider? *Check the box that best suits your situation.*

|  | Strongly agree | Agree | Neutral | Disagree | Strongly disagree |
| --- | --- | --- | --- | --- | --- |
| I could easily get the test | □ | □ | □ | □ | □ |
| I felt comfortable | □ | □ | □ | □ | □ |
| It affected me emotionally | □ | □ | □ | □ | □ |
| I was embarrassed | □ | □ | □ | □ | □ |
| I experienced difficulties | □ | □ | □ | □ | □ |
| I got good instructions | □ | □ | □ | □ | □ |
| I thought there was (too) little anonymity | □ | □ | □ | □ | □ |

*The following questions are about performing the HIV self-test (or combination HIV/STD self-test)*

1. Have you done the HIV self-test (or combination HIV/STD self-test)?

*Note: you do not have to share your test result, if you do not want to.*

- No (not yet)
- Yes

1. [If Q25 = no] Why have you not (yet) done the HIV self-test (or combination HIV/STD self-test)? [compulsory question]

- I didn’t have the time yet
- I forgot
- I don’t think I have an HIV infection
- I will wait until there is a reason to test myself
- I will give the test to someone else
- I am nervous to do the test
- I already took an HIV test through a healthcare provider (GGD/GP)
- Other, namely

[if Q25 = yes] continue questionnaire

[if Q25 = no] skip to question 32

1. Did you use the assisted option of HIV self-testing? [for community provider only]

- No
- Yes

1. What information did you use when you did the HIV self-test (or combination HIV/STD self-test)? *Multiple answers are possible.*

□ Through communication with a counselor via WhatsApp [for community provider only]

□ Option of assisted testing when the procedure is explained by the community provider [for community provider only]

□ Information on the website of the current provider

□ The paper manual that came with the HIV test

□ Instruction videos on the website of the current provider

□ Instruction videos on another website

□ I have not used any information

□ Other, namely

1. Have you experienced any problems with performing the HIV self-test (or combination HIV/STD self-test)?

- No
- Yes

1. [if Q29 = yes] What problem(s) did you experience performing the HIV self-test? M*ultiple answers are possible.*
   - The steps for performing the test were too complicated
   - The instructions along with the test were not clear to me
   - Part of the test package was missing
   - I forgot one step when performing the test
   - I am afraid of performing the finger prick to myself
   - I am afraid of blood with the finger prick
   - The finger prick was too hard to do, there was not enough blood coming from my finger
   - There was no result shown / the result was unclear
   - I didn’t know how to read the test result
   - Other, namely

*The last questions are about the result of your HIV self-test (or combination HIV/STD self-test)*

1. What was the result of the HIV self-test that you bought/received from the current provider? *Note: this questionnaire is entirely anonymous and your answer will not be shared.*

- HIV infection (HIV positive)
- No HIV infection (HIV negative)
- I decided not to look for the results
- The test failed
- I would rather not say

1. Would you know what to do if you got an HIV positive self-test result?

- No
- Yes
- I would rather not say

*These are the final questions about the services of the current provider and the accessibility of HIV self-tests.*

1. Overall, what was your experience of the service of the current provider?

- Satisfied
- Neutral
- Non-satisfied

Please clarify:

1. What recommendations do you have to improve the information and/or service of the current provider? *Open question.*
2. What recommendations do you have to improve the accessibility of HIV self-tests?

*Open question.*

*You have reached the end of the questionnaire.*

**Supplementary Table S2.** Characteristics of study participants using HIVSS/ST by sexual orientation in the Netherlands

|  |  | Women and heterosexual men | GBM | Test statistic | |
| --- | --- | --- | --- | --- | --- |
|  |  | n (%) | n (%) | X^2^ (df) ^a^ | p-value |
| Total | | 80 (100) | 49 (100) |  |  |
| organization | |  |  | 1.434 (1) | 0.231 |
|  | Community-based provider | 29 (36.2) | 12 (4.5) |  |  |
|  | Online commercial provider | 51 (63.8) | 37 (75.5) |  |  |
| Age group | |  |  | 4.639 (2) | 0.098 |
|  | 18-24 | 11 (13.8) | 13 (26.5) |  |  |
|  | 25-34 | 41 (51.2) | 17 (34.7) |  |  |
|  | 35+ | 28 (35.0) | 19 (38.8) |  |  |
| Country of residence | |  |  | - | 0.525 |
|  | The Netherlands | 78 (97.5) | 49 (100.0) |  |  |
|  | Other | 2 (2.5) | 0 (0.0) |  |  |
| Province of residence^b^ | |  |  | - | 0.182 |
|  | North (Drenthe, Groningen, Friesland) | 5 (6.4) | 2 (4.1) |  |  |
|  | East (Flevoland, Overijssel, Gelderland) | 6 (7.7) | 10 (20.4) |  |  |
|  | South (Noord-Brabant, Limburg) | 14 (17.9) | 10 (20.4) |  |  |
|  | West (Noord-Holland, Zuid-Holland, Utrecht, Zeeland) | 53 (67.9) | 27 (55.1) |  |  |
| Place of residence^b^ | |  |  | 2.132 (1) | 0.144 |
|  | City | 60 (76.9) | 31 (63.3) |  |  |
|  | Village | 18 (23.1) | 18 (36.7) |  |  |
| Country of birth | |  |  | 0.368 (1) | 0.544 |
|  | The Netherlands | 50 (62.5) | 34 (69.4) |  |  |
|  | Other | 30 (37.5) | 15 (30.6) |  |  |
| Education level | |  |  | 0.817 (1) | 0.366 |
|  | ≥Higher vocational education or University | 64 (80.0) | 35 (71.4) |  |  |
|  | ≤Intermediate vocational education | 16 (20.0) | 14 (28.6) |  |  |
| Relationship status | |  |  | - | 0.063 |
|  | Single | 26 (32.5) | 23 (46.9) |  |  |
|  | Dating | 34 (42.5) | 10 (20.4) |  |  |
|  | Steady partner | 17 (21.2) | 13 (26.5) |  |  |
|  | Other | 3 (3.8) | 3 (6.1) |  |  |
| Type HIV test | |  |  | 0.902 (1) | 0.342 |
|  | HIV self-test | 29 (36.3) | 13 (26.5) |  |  |
|  | HIV self-sample | 51 (63.7) | 36 (73.5) |  |  |

Gender-diverse persons were excluded from these analyses. *GBM* Gay and Bisexual Men

^a^ If more than 20% of the expected cell counts were below 5, Fisher’s exact test was applied instead of chi-square test.

^b^ Percentage of persons living in the Netherlands.

**Supplementary Table S3.** Characteristics of study participants using HIVSS/ST first-time and repeatedly in the Netherlands

|  |  | First-time users | Repeat users | Test statistic | |
| --- | --- | --- | --- | --- | --- |
|  |  | n (%) | n (%) | X^2^ (df) ^a^ | p-value |
| Total | | 44 (100) | 89 (100) |  |  |
| Organization | |  |  | 0.001 (1) | 0.982 |
|  | Community-based provider | 14 (31.8) | 30 (33.7) |  |  |
|  | Online commercial provider | 30 (68.2) | 59 (66.3) |  |  |
| Gender and sexual orientation | |  |  | **-** | **0.007** |
|  | Women | 19 (43.2) | 28 (31.5) |  |  |
|  | Heterosexual men | 16 (36.4) | 17 (19.1) |  |  |
|  | GBM | 8 (18.2) | 41 (46.1) |  |  |
|  | Transgender/non-binary/other | 1 (2.3) | 3 (3.4) |  |  |
| Age group | |  |  | 2.960 (2) | 0.228 |
|  | 18-24 | 12 (27.3) | 14 (15.7) |  |  |
|  | 25-34 | 19 (43.2) | 39 (43.8) |  |  |
|  | 35+ | 13 (29.5) | 36 (40.4) |  |  |
| Country of residence | |  |  | - | 1.000 |
|  | The Netherlands | 44 (100.0) | 87 (97.8) |  |  |
|  | Other | 0 (0.0) | 2 (2.2) |  |  |
| Place of residence in the Netherlands^b^ | | |  | - | 0.604 |
|  | North (Drenthe, Groningen, Friesland) | 1 (2.3) | 6 (6.9) |  |  |
|  | East (Flevoland, Overijssel, Gelderland) | 7 (15.9) | 9 (10.3) |  |  |
|  | South (Noord-Brabant, Limburg) | 9 (20.5) | 16 (18.4) |  |  |
|  | West (Noord-Holland, Zuid-Holland, Utrecht, Zeeland) | 27 (61.4) | 56 (64.4) |  |  |
| Place of residence^b^ | |  |  | 0.000 (1) | 1.000 |
|  | City | 32 (72.7) | 63 (72.4) |  |  |
|  | Village | 12 (27.3) | 24 (27.6) |  |  |
| Country of birth | |  |  | 0.057 (1) | 0.812 |
|  | The Netherlands | 27 (61.4) | 58 (65.2) |  |  |
|  | Other | 17 (38.6) | 31 (34.8) |  |  |
| Education level | |  |  | 0.395 (1) | 0.530 |
|  | ≥Higher vocational education or University | 36 (81.8) | 67 (75.3) |  |  |
|  | ≤Intermediate vocational education | 8 (18.2) | 22 (24.7) |  |  |
| Relationship status | |  |  | - | 0.115 |
|  | Single | 21 (47.7) | 29 (32.6) |  |  |
|  | Dating | 16 (36.4) | 31 (34.8) |  |  |
|  | Steady partner | 7 (15.9) | 23 (25.8) |  |  |
|  | Other | 0 (0.0) | 6 (6.7) |  |  |
| Type HIV test | |  |  | 0.023 (1) | 0.880 |
|  | HIV self-test | 14 (31.8) | 31 (34.8) |  |  |
|  | HIV self-sample | 30 (68.2) | 58 (65.2) |  |  |

*GBM* Gay and Bisexual men

^a^ If more than 20% of the expected cell counts were below 5, Fisher’s exact test was applied instead of chi-square test.

^b^ Percentage of persons living in the Netherlands.

**Supplementary Table S4.** HIV-related characteristics of study participants using HIVSS/ST by sexual orientation in the Netherlands

|  |  | Women and heterosexual men | GBM | Test statistic | |
| --- | --- | --- | --- | --- | --- |
|  |  | n (%) | n (%) | X^2^ (df) ^a^ | p-value |
| Total | | 80 (100) | 49 (100) |  |  |
| HIV test result | |  |  | - | 0.407 |
|  | HIV negative | 75 (93.8) | 48 (98.0) |  |  |
|  | HIV positive | 0 (0.0) | 0 (0.0) |  |  |
|  | Unknown^b^ | 5 (6.2) | 1 (2.0) |  |  |
| Ever tested for HIV | |  |  | 28.314 (3) | **<0.001** |
|  | No | 35 (43.8) | 8 (16.3) |  |  |
|  | Yes, 1-2 times | 32 (40.0) | 14 (28.6) |  |  |
|  | Yes, 3-4 times | 8 (10.0) | 6 (12.2) |  |  |
|  | Yes, 5 times or more | 5 (6.2) | 21 (42.9) |  |  |
| When was last HIV test?^c^ | |  |  | **-** | **0.002** |
|  | In the last 6 months | 16 (35.6) | 23 (56.1) |  |  |
|  | 6-12 months ago | 5 (11.1) | 11 (26.8) |  |  |
|  | More than 1 year ago | 23 (51.1) | 7 (17.1) |  |  |
|  | I don't remember | 1 (2.2) | 0 (0.0) |  |  |
| Ever used PrEP | |  |  | **-** | **<0.001** |
|  | No | 80 (100.0) | 40 (81.6) |  |  |
|  | Yes | 0 (0.0) | 9 (18.4) |  |  |
| Condomless sex past 3 months | |  |  | - | 0.329 |
|  | No | 18 (22.5) | 16 (32.7) |  |  |
|  | Yes | 60 (75.0) | 33 (67.3) |  |  |
|  | I would rather not say | 2 (2.5) | 0 (0.0) |  |  |
| Condomless sex last intercourse | |  |  | - | 0.051 |
|  | No | 23 (28.8) | 23 (46.9) |  |  |
|  | Yes | 54 (67.5) | 26 (53.1) |  |  |
|  | I would rather not say | 3 (3.8) | 0 (0.0) |  |  |
| Type of HIVSS/ST^d^ | |  |  | **-** | **0.009** |
|  | Combination STI SS package (Chlamydia, Gonorrhea, (Trichomoniasis), syphilis and HIV) | 29 (56.9) | 10 (27.0) |  |  |
|  | Syphilis- and HIV-SS | 11 (21.6) | 8 (21.6) |  |  |
|  | HIVSS | 11 (21.6) | 18 (48.6) |  |  |
|  | HIVST | 0 (0.0) | 1 (2.7) |  |  |
| How obtained HIVSS/ST^d^ | |  |  | - | 0.366 |
|  | Package delivery | 4 (7.8) | 5 (13.5) |  |  |
|  | Letter post delivery | 36 (70.6) | 25 (67.6) |  |  |
|  | Pick-up at package point | 9 (17.6) | 3 (8.1) |  |  |
|  | Pick-up at regular pharmacy (not online) | 2 (3.9) | 3 (8.1) |  |  |
|  | Pick-up at 24-hour vending machine | 0 (0.0) | 1 (2.7) |  |  |
| Performed HIVSS/ST | |  |  | - | 0.288 |
|  | No | 3 (3.8) | 0 (0.0) |  |  |
|  | Yes | 77 (96.2) | 49 (100.0) |  |  |
| If not, why | |  |  | - | 1.000 |
|  | I didn’t have the time yet | 2 (66.7) | 0 (NA) |  |  |
|  | I forgot | 1 (33.3) | 0 (NA) |  |  |
| Problems performing HIVSS/ST^e^ | |  |  | 3.174 (1) | 0.075 |
|  | No | 56 (72.7) | 43 (87.8) |  |  |
|  | Yes | 21 (27.3) | 6 (12.2) |  |  |
| Would you know what to do if you got an HIV positive ST? | |  |  | - | 0.243 |
|  | No | 25 (31.2) | 15 (30.6) |  |  |
|  | Yes | 55 (68.8) | 32 (65.3) |  |  |
|  | I would rather not say | 0 (0.0) | 2 (4.1) |  |  |

Gender-diverse persons were excluded from these analyses.

*GBM* Gay and Bisexual men, *SS* self-sample, *ST* self-test, *STI* sexually transmitted infections, *NA* not available.

^a^ If more than 20% of the expected cell counts were below 5, Fisher’s exact test was applied instead of chi-square test.

^b^ Unknown include persons who would rather not say the result, have not looked for the results or have not taken the test yet and persons of whom the test failed.
^c^ Percentage of persons ever tested for HIV.

^d^ Only included in questionnaire of commercial provider; percentage of persons at commercial provider.
^e^ Percentage of persons who performed the HIVSS/ST.

**Supplementary Table S5.** HIV-related characteristics of study participants using HIVSS/ST first-time and repeatedly in the Netherlands

|  |  | First-time users | Repeat users | Test statistic | |
| --- | --- | --- | --- | --- | --- |
|  |  | n (%) | n (%) | X^2^ (df) ^a^ | p-value |
| Total | | 44 (100) | 89 (100) |  |  |
| HIV test result | |  |  | - | 1.000 |
|  | HIV negative | 42 (95.5) | 85 (95.5) |  |  |
|  | HIV positive | 0 (0.0) | 0 (0.0) |  |  |
|  | Unknown^b^ | 2 (4.5) | 4 (4.5) |  |  |
| Ever used PrEP | |  |  | **-** | **0.029** |
|  | No | 44 (100.0) | 80 (89.9) |  |  |
|  | Yes | 0 (0.0) | 9 (10.1) |  |  |
| Condomless sex past 3 months | |  |  | - | 0.218 |
|  | No | 16 (36.4) | 21 (23.6) |  |  |
|  | Yes | 27 (61.4) | 67 (75.3) |  |  |
|  | I would rather not say | 1 (2.2) | 1 (1.1) |  |  |
| Condomless sex last intercourse | |  |  | - | 0.432 |
|  | No | 16 (36.4) | 32 (36.0) |  |  |
|  | Yes | 26 (59.1) | 56 (62.9) |  |  |
|  | I would rather not say | 2 (4.5) | 1 (1.1) |  |  |
| Type of HIVSS/ST^c^ | |  |  | - | 0.064 |
|  | Combination STI SS package (Chlamydia, Gonorrhea, (Trichomoniasis), syphilis and HIV) | 18 (60.0) | 22 (37.3) |  |  |
|  | Syphilis- and HIV-SS | 7 (23.3) | 12 (20.3) |  |  |
|  | HIVSS | 5 (16.7) | 24 (40.7) |  |  |
|  | HIVST | 0 (0.0) | 1 (1.7) |  |  |
| How obtained HIVSS/ST^c^ | |  |  | - | 0.430 |
|  | Package delivery | 3 (10.0) | 6 (10.2) |  |  |
|  | Letter post delivery | 21 (70.0) | 40 (67.8) |  |  |
|  | Pick-up at package point | 6 (20.0) | 7 (11.9) |  |  |
|  | Pick-up at regular pharmacy (not online) | 0 (0.0) | 5 (8.5) |  |  |
|  | Pick-up at 24-hour vending machine | 0 (0.0) | 1 (1.7) |  |  |
| Performed HIVSS/ST | |  |  | - | 1.000 |
|  | No | 1 (2.3) | 2 (2.2) |  |  |
|  | Yes | 43 (97.7) | 87 (97.8) |  |  |
| If not, why | |  |  | - | 0.333 |
|  | I didn’t have the time yet | 0 (0.0) | 2 (100.0) |  |  |
|  | I forgot | 1 (100.0) | 0 (0.0) |  |  |
| Problems performing HIVSS/ST^d^ | |  |  | 0.012 (1) | 0.914 |
|  | No | 33 (76.7) | 69 (79.3) |  |  |
|  | Yes | 10 (23.3) | 18 (20.7) |  |  |
| Would you know what to do if you got an HIV positive ST? | |  |  | - | 0.111 |
|  | No | 15 (34.1) | 26 (29.2) |  |  |
|  | Yes | 27 (61.4) | 63 (70.8) |  |  |
|  | I would rather not say | 2 (4.5) | 0 (0.0) |  |  |

*SS* self-sample, *ST* self-test, *STI* sexually transmitted infections.

^a^ If more than 20% of the expected cell counts were below 5, Fisher’s exact test was applied instead of chi-square test.

^b^ Unknown include persons who would rather not say the result, have not looked for the results or have not taken the test yet and persons of whom the test failed.
^c^ Only included in questionnaire of commercial provider.
^d^ Only included in questionnaire of commercial provider; percentage of persons at commercial provider.

**Supplementary Table S6.** Usability and preferences of study participants using HIVSS/ST by sexual orientation in the Netherlands

|  |  | Women and heterosexual men | GBM | Test statistic | |
| --- | --- | --- | --- | --- | --- |
|  |  | n (%) | n (%) | X^2^ (df) ^a^ | p-value |
| Total | | 80 (100) | 49 (100) |  |  |
| How did you hear about HIVSS/ST? | |  |  |  |  |
|  | Pharmacy | 0 (0.0) | 0 (0.0) | - | - |
|  | GP | 1 (1.3) | 0 (0.0) | - | 1.000 |
|  | Public health services (GGD) or SHC | 3 (3.8) | 9 (18.4) | **-** | **0.010** |
|  | Previous HIVSS/ST | 11 (13.8) | 8 (16.3) | 0.021 (1) | 0.885 |
|  | A friend | 8 (10.0) | 1 (2.0) | - | 0.152 |
|  | Internet | 62 (77.5) | 37 (75.5) | 0.002 (1) | 0.964 |
|  | Social media | 1 (1.3) | 1 (2.0) | - | 1.000 |
| Main reasons for HIVSS/ST at community-based or online commercial provider | |  |  | |  |
|  | It is cheap^b^ | 8 (10.0) | 2 (4.1) | - | 0.317 |
|  | It is for free^c^ | 27 (33.8) | 12 (24.5) | 0.835 (1) | 0.361 |
|  | Good previous experiences with online order of HIV or STI self-tests | 9 (11.3) | 6 (12.2) | 0.000 (1) | 1.000 |
|  | Good previous experiences with this commercial test provider^b^ | 14 (17.5) | 11 (22.4) | 0.212 (1) | 0.645 |
|  | Good previous experiences with this community provider^c^ | 4 (5.0) | 1 (2.0) | - | 0.649 |
|  | Does not require to talk to my GP about HIV tests | 28 (35.0) | 18 (36.7) | 0.000 (1) | 0.992 |
|  | I don’t have a GP | 6 (7.5) | 4 (8.2) | - | 1.000 |
|  | Anonymity | 31 (38.8) | 16 (32.7) | 0.260 (1) | 0.610 |
|  | No GGD/SHC nearby | 4 (5.0) | 7 (14.3) | - | 0.102 |
|  | No (soon) open spots available at GGD/SHC | 19 (23.8) | 12 (24.5) | 0.000 (1) | 1.000 |
|  | Don’t know any other way to test for HIV | 4 (5.0) | 4 (8.2) | - | 0.478 |
|  | Prefer a finger prick test, because I don’t want blood drawn | 3 (3.8) | 2 (4.1) | - | 1.000 |
|  | Immediate results | 21 (26.3) | 11 (22.4) | 0.076 (1) | 0.783 |
|  | It saves time | 39 (48.8) | 16 (32.7) | 2.595 (1) | 0.110 |
| Information used with performing HIVSS/ST^d^ | |  |  |  |  |
|  | On website of test provider | 16 (20.8) | 11 (22.4) | 0.000 (1) | 1.000 |
|  | The paper manual that came with the HIV test | 70 (90.9) | 46 (93.9) | - | 0.739 |
|  | Instruction videos on website of test provider | 18 (23.4) | 7 (14.3) | 1.037 (1) | 0.309 |
|  | Instruction videos on another website | 4 (5.2) | 2 (4.1) | - | 1.000 |
|  | No information used | 1 (1.3) | 0 (0.0) | - | 1.000 |
| If experienced problems performing HIVSS/ST, what problems^e^ | |  |  |  |  |
|  | Steps for performing the test too complicated | 0 (0.0) | 0 (0.0) | - | - |
|  | Instructions with test were not clear | 4 (19.0) | 0 (0.0) | - | 0.545 |
|  | Forgot one step when performing test | 0 (0.0) | 0 (0.0) | - | - |
|  | Afraid of performing finger prick | 6 (28.6) | 0 (0.0) | - | 0.284 |
|  | Afraid of blood with finger prick | 0 (0.0) | 0 (0.0) | - | - |
|  | Not enough blood from finger prick | 14 (66.7) | 5 (83.3) | - | 0.633 |
|  | No results / unclear test result | 1 (4.8) | 0 (0.0) | - | 1.000 |
|  | Don’t know how to read the test result | 1 (4.8) | 0 (0.0) | - | 1.000 |

*GBM* Gay and Bisexual men, *GP* general practitioner, *SHC* Sexual Health Center.

^a^ If more than 20% of the expected cell counts were below 5, Fisher’s exact test was applied instead of chi-square test.

^b^ Only included in questionnaire of commercial provider.

^c^ Only included in questionnaire of community-based provider.

^d^ Percentage of persons who performed the HIVSS/ST.

^e^ Percentage of persons who performed the HIVSS/ST and experienced problems.

**Supplementary Table S7.** Usability and preferences of study participants using HIVSS/ST first-time and repeatedly in the Netherlands

|  |  | First-time users | Repeat users | Test statistic | | |
| --- | --- | --- | --- | --- | --- | --- |
|  |  | n (%) | n (%) | X^2^ (df) ^a^ | | p-value |
| Total | | 44 (100) | 89 (100) |  | |  |
| How did you hear about HIVSS/ST? | |  |  |  | |  |
|  | Pharmacy | 0 (0.0) | 0 (0.0) | - | | - |
|  | GP | 1 (2.3) | 0 (0.0) | - | | 0.331 |
|  | Public health services (GGD) or SHC | 2 (4.5) | 10 (11.2) | - | | 0.336 |
|  | Previous HIVSS/ST | 2 (4.5) | 17 (19.1) | 3.975 (1) | | **0.046** |
|  | A friend | 3 (6.8) | 8 (9.0) | - | | 1.000 |
|  | Internet | 37 (84.1) | 64 (71.9) | 1.771 (1) | | 0.183 |
|  | Social media | 0 (0.0) | 2 (2.2) | - | | 1.000 |
| Main reasons for HIVSS/ST at community-based or online commercial provider | |  | | | | |
|  | It is cheap^b^ | 4 (9.1) | 6 (6.7) | - | | 0.730 |
|  | It is for free^c^ | 13 (29.5) | 29 (32.6) | 0.024 (1) | | 0.876 |
|  | Good previous experiences with online order of HIV or STI self-tests | 4 (9.1) | 12 (13.5) | 0.202 (1) | | 0.654 |
|  | Good previous experiences with this commercial test provider^b^ | 5 (11.4) | 20 (22.5) | 1.708 (1) | | 0.191 |
|  | Good previous experiences with this community provider^c^ | 1 (2.3) | 4 (4.5) | - | | 1.000 |
|  | Does not require to talk to my GP about HIV tests | 14 (31.8) | 33 (37.1) | 0.164 (1) | | 0.686 |
|  | I don’t have a GP | 4 (9.1) | 6 (6.7) | - | | 0.730 |
|  | Anonymity | 19 (43.2) | 29 (32.6) | 1.011 (1) | | 0.315 |
|  | No GGD/SHC nearby | 2 (4.5) | 9 (10.1) | - | | 0.337 |
|  | No (soon) open spots available at GGD/SHC | 10 (22.7) | 23 (25.8) | 0.032 (1) | | 0.859 |
|  | Don’t know any other way to test for HIV | 4 (9.1) | 4 (4.5) | - | | 0.439 |
|  | Prefer a finger prick test, because I don’t want blood drawn | 3 (6.8) | 2 (2.2) | - | | 0.331 |
|  | Immediate results | 11 (25.0) | 23 (25.8) | 0.000 (1) | | 1.000 |
|  | It saves time | 20 (45.5) | 36 (40.4) | 0.132 (1) | | 0.716 |
| Information used with performing HIVSS/ST^d^ | |  |  |  | |  |
|  | On website of test provider | 9 (20.9) | 19 (21.8) | 0.000 (1) | | 1.000 |
|  | The paper manual that came with the HIV test | 36 (83.7) | 83 (95.4) | **-** | | **0.040** |
|  | Instruction videos on website of test provider | 10 (23.3) | 15 (17.2) | 0.339 (1) | | 0.561 |
|  | Instruction videos on another website | 2 (4.7) | 5 (5.7) | - | | 1.000 |
|  | No information used | 1 (2.3) | 0 (0.0) | - | | 0.331 |
| If experienced problems performing HIVSS/ST, what problems^e^ | |  |  | |  | |
|  | Steps for performing the test too complicated | 0 (0.0) | 0 (0.0) | - | | - |
|  | Instructions with test were not clear | 1 (10.0) | 3 (16.7) | - | | 1.000 |
|  | Forgot one step when performing test | 0 (0.0) | 0 (0.0) | - | | - |
|  | Afraid of performing finger prick | 3 (30.0) | 3 (16.7) | - | | 0.634 |
|  | Afraid of blood with finger prick | 0 (0.0) | 0 (0.0) | - | | - |
|  | Not enough blood from finger prick | 8 (80.0) | 12 (66.7) | - | | 0.669 |
|  | No results / unclear test result | 0 (0.0) | 1 (5.6) | - | | 1.000 |
|  | Don’t know how to read the test result | 0 (0.0) | 1 (5.6) | - | | 1.000 |

*GP* general practitioner, *SHC* Sexual Health Center.

^a^ If more than 20% of the expected cell counts were below 5, Fisher’s exact test was applied instead of chi-square test.

^b^ Only included in questionnaire of commercial provider.

^c^ Only included in questionnaire of community-based provider.

^d^ Percentage of persons who performed the HIVSS/ST.

^e^ Percentage of persons who performed the HIVSS/ST and experienced problems.
